# Incorporating dialogue in laboratory teaching

**DOI:** 10.1007/s00216-024-05569-2

**Published:** 2024-10-01

**Authors:** Michael K. Seery, Hendra Y. Agustian, Frederik V. Christiansen, Bente Gammelgaard, Rie H. Malm

**Affiliations:** 1 Centre for Academic Language and Development, University of Bristol, Bristol, UK; 2 Department of Science Education, Faculty of Science, University of Copenhagen, Copenhagen, Denmark; 3 Department of Pharmacy, Faculty of Health and Medical Sciences, University of Copenhagen, Copenhagen, Denmark

## Introduction

This “Education and Professional Development” article aims to encourage those who teach in laboratories to consider how to incorporate dialogue into laboratory activity. Within the context of the importance of dialogue in learning — both from a theoretical perspective as well as the literature on laboratory teaching in practice — the article sets out some practical examples about how a culture of dialogue can be embedded into laboratory teaching using three examples. To assist educators in thinking about dialogue in their own practice, some prompts relevant to analytical chemistry are provided. Overall, the article asserts the valuable outcomes associated with ensuring the explicit role of dialogue in laboratory teaching.

One of the many benefits of the laboratory environment in chemistry is that it provides a more relaxed and informal space for students to engage in their studies. The format facilitates dialogue and connection with between students and instructors — professors and graduate teaching assistants alike — enabling students to feel part of a community of chemists. This time is highly valued by students, as it means they can readily access expertise to support their learning, as well as allowing instructors to gain insights about their students [1–3].

However, observations of laboratory teaching in practice indicate that dialogue and opportunity for meaningful discussions may not happen organically. While this may be because students’ focus is on completing the task at hand in an efficient a manner as possible [4], follow-up individual discussions by email, as well as feedback from students that writing up laboratory work takes excessive amounts of time, suggest that there is benefit from having opportunity to discuss student work more during laboratory time [5]. Therefore, we argue that there is a benefit to incorporating dialogue more formally into laboratory teaching practices in ways that engage students in meaningful conversations about their work, and how they can begin and continue its analysis both in the laboratory time and in study time after the laboratory. As well as providing a means to support the known challenge of writing laboratory reports [6], this intends to provide an additional benefit: an opportunity for students to *talk* about chemistry and laboratory work more in practice, rather than just *write* about it. This is an important skill for students to develop, so that they are able to talk about their work in a meaningful way in post-graduation interviews, workplace, and society.

One form of dialogue that is more common in laboratory teaching is where assessment includes oral components, such as an oral examination [7, 8] or presentation of findings or related concepts [9, 10]. In these cases, dialogue through the form of oral assessment is intended to diversify the assessment matrix to consider a broader range of intended learning outcomes. We shift our emphasis on dialogue in our discussion below to focus on dialogue for formative rather than for assessment reasons; that is to say, dialogue *for* learning.

### Why is dialogue important?

Laboratories are social spaces in which students and teachers share the common goal of learning about laboratory techniques and the process of doing science [11]. In her work on dialogue in school practical environments, Hennah describes the importance of centralising dialogue in these laboratory spaces as the means by which students will challenge current understanding and create new (to them) knowledge [12], pointing to the benefit of introducing “talk moves” for formalising the role of dialogue. Talk moves are described by Hennah as ways in which specific responses can be used to further encourage students’ “academically productive talk”, such as prompts to link knowledge or to evaluate a particular result. Christiansen and Olsen [13] have used Brousseau’s theory of didactic situations which emphasises interactions to consider the importance of different stages in mediating learning in an environment. These include (i) formulation: enabling space for students to express themselves and create hypotheses about their work; (ii) validation: testing those hypotheses either with other students or the teacher; and (iii) institutionalisation: engaging in dialogue with the teacher to consider the broader context of their findings. These stages map on well to the kinds of settings available in laboratory teaching environments, and hence point to a valuable opportunity to incorporate dialogue into laboratory teaching in a manner that is conducive to learning.

As well as theoretical framing, dialogue has played a central role in innovative laboratory practice as reported in the empirical literature over the last decade. A review of over 150 publications in chemistry education synthesised to develop “10 Guiding Principles” led to the inclusion of “incorporating dialogue” as one of the ten principles [14]. Laboratory activities that centralise on discussion have also been shown to improve students’ sense of capacity to do well in those settings [15], and indeed in helping students’ development in scientific thinking more generally. More explicit inclusion of dialogue helps students develop “thinking as scientist” skills as dialogue in scientific context is modelled by instructors in their interaction with students. Given the propensity of assessment of laboratory work towards written assessment, incorporation of dialogue in the broadest sense offers an opportunity for students to build confidence and speak about scientific concepts in a structured and relatively informal environment [16]. As described below, it is this formative aspect that we consider the most important benefit for learning.

### Examples of formalising dialogue in laboratory teaching

The intention of this article is to describe some of the ways in which we have implemented this principle in a variety of contexts. We describe examples of how dialogue can be incorporated more formally into laboratory teaching, drawing from our own practice and related literature. We’ve chosen these particular examples as ones having the most impact on the laboratory learning environment in terms of supporting student engagement and learning. Particular contexts of some of these approaches have been described elsewhere [3, 5, 17, 18]; our purpose is here to consider specifically the lens of dialogue and how it can be incorporated across a range of contexts. The primary message is that these activities are embedded into the curriculum delivery as an explicit part of the laboratory experience — they are a required part of the teaching and learning process in which students should engage, so as to help contribute to their understanding.

### Example 1: Discussion with students at the beginning of laboratory session about some key concepts relating to laboratory work

Establishing a culture of normalising dialogue in the laboratory class requires students having availability and capacity to draw on related theory and concepts so that they can make some informed discussions. Typical approaches are the use of pre-laboratory activities to introduce students to some of the overarching substantive concepts to enable meaningful insights and discussion on these concepts to be incorporated into laboratory time. These include both the chemical concepts relating to the experiment and understanding of the experimental approach [19]. A common intention of educators is that students will draw on pre-laboratory work and information in the laboratory manual to inform their in-laboratory activities. However, without checking, the extent to which students have engaged in these activities is uncertain, and consequently, there is a lack of insight into any areas of difficulties for students about these concepts. In the absence of any formal discussion prompts, it is left to students to ask about difficulties. If they do not, these difficulties do not become apparent until after the laboratory report has been completed.

In our practice, formalised discussion prompts at the start of the laboratory class are used as a bridge to connect preparation activities with in-laboratory work [5]. Some example prompts for a laboratory session involving instrumentation (UV/vis spectrometer) as shown in Table 1. These prompts were provided to laboratory instructors to use as a basis for discussion in the start-up phase of the laboratory. As students begin their experiment, this structured dialogue focussed on ensuring that they were able to share some conceptual understandings and any difficulties early on in the laboratory. This tends to take the form of Socratic dialogue rather than direct “testing” of students, with the aim that students are prompted to think through the rationale for various parameters involved in experimental setup. It allows for students to draw on what they have been presented with in their pre-laboratory information and the laboratory manual in a manner that conveys a culture valuing discussion. In our iterations, this tended to happen with the instructor discussing with each group of 2–3 students doing an experiment over the first 15–20 min of a laboratory session, but could work equally well with small clusters of groups, with the instructor ensuring that all participants are engaged in dialogue. The intention at the end of this dialogue session is that students are able to articulate more of the rationale underpinning various experimental parameters of the work they are about to do, such as suitability of instrument/technique for intended purpose and experimental parameters (e.g. settings or solvent choice).

**Table 1:** Example prompts for students at the start of a laboratory practical using instrumental technique (in this case UV/visible spectroscopy) focussing on experimental method, parameters, and materials

Prompt	Rationale for prompt
Why is this technique useful for the purpose of this experiment?	Prompting thinking on experimental method and relationship with theory (e.g. Beer Lambert Law); follow-on prompts may relate to whether there is a colour change (e.g. in kinetics) and how the technique will be valuable for that purpose
What wavelength range/value is appropriate for this experiment?	Prompting thinking on experimental parameters and relationship with chemical substances they are working with; follow-on prompts may relate to thinking about colour/lack of colour
Why should we use a [quartz/glass/PMMA] cell for this experiment?	Prompting thinking on experimental materials; tasked in the moment will help students gain greater insight into the experimental actions taken and what informs the decisions to take them

These prompts build on the pre-laboratory activities students are tasked to complete, and are used primarily for two purposes. The first is to assert the importance of the preparation work and its role in the laboratory, as students know that to engage meaningfully in the preliminary discussions, they will need to complete this work in advance. The second is to set the stage for dialogue early on in the laboratory session and, importantly, the role of dialogue in supporting learning. This means that the discussions at the start of the session should not be seen as any form of assessment, but rather as a place where students can check their understanding and areas of difficultly, seeding the interactions for the remainder of the laboratory class. This kind of approach can be extended by structuring in-laboratory peer review dialogue, as previously described for laboratory skills by Musgrove [20].

### Example 2: Laboratory manual contains prompted decisions which require discussion among students and agreement of approach

The highly structured nature of most laboratory activities mean that students will tend to follow the laboratory manual as their main guide for completion of laboratory work. This provides for a convenient way to incorporate dialogue prompts throughout the laboratory teaching session, by including prompts in the manual. These can be used to prompt students to make meaning of experimental results and the conclusions they can draw in relation to their hypothesis as they conduct the experiment. The intention here is for students to consider their experimental data in context, during the laboratory time, to help them make sense of the meaning of this data as it relates to the purpose of the experiment. By providing laboratory instructors with more specific dialogue prompts, it helps formalise the mid-laboratory discussion activity and move it beyond a general check-in that we commonly observed in our practice (typified by generic-type statements such as “how is everything going?”).

This can be achieved in various ways. In our implementation, dialogue prompts in the laboratory manual were used as signals to students to check in with their instructor, and because different students tend to move through the laboratory at different rates. If students complete experiments on rotation, these conversations tend to happen with individual groups of students and the laboratory instructor responsible for a particular experiment. However, if instructor to student ratio required it, these discussions could be more formalised, with a mid-experiment check-in stage built into the laboratory timing, so that groups of students can engage in the discussion at once. Overall, the intention is to move the dialogue on from the earlier conversation about overarching concepts, and on towards thinking about how the results obtained relate to these concepts. Depending on the stage of the experiment, they can ask students to make judgements at points in the experiment where they have to make a decision, and prompt discussion with peers and teacher about their rationale for those decisions. Some examples of prompts useful for a chromatography laboratory are shared in Table 2.

**Table 2:** Example prompts for students when discussing intermediate stage results to determine the next steps to take in experimental work

Prompt	Rationale
What features are we looking for in the [GC/HPLC] output when determining the suitability of an eluent?	Prompting thought on parameters on which judgements will be made, so that experimental information being discussed can be applied to those parameters in a clear way
What eluent component ratios do we think were most useful from our shared data?	Prompting thought on experimental results obtained, and making claims based on the evidence available. Follow on prompts can consider, for example, the role of pH or polarity
What are the considerations for using this eluent for our next phase of analysis?	Prompting thought on hypothesis in the next stage of experimental work — for example there may be issues relating to pH stability, or time-stability considerations

This kind of approach works well when the experiment design builds in decision-making points in the laboratory, for example by including an optimisation phase (such as tasking students with different ratios of eluent components for a particular chromatographic technique), and then sharing results in the class to foster discussions about which particular ratio would be optimal for a subsequent analysis. Results sharing can take the form of a pooled spreadsheet with summary analysis/results available for discussion among the group [21]: the focus of a shared sheet provides a useful structure for student activity [12]. Discussion prompts here should aim to integrate student ideas, making clear the considerations required to draw conclusions from scientific data.

In practice, such prompts can become more difficult for students to know what to talk about, as they are required to move on from thinking about experimental principles discussed earlier to thinking about how their results can be considered in light of the framing theory. Thus, discussions at this stage need to be managed to support students thinking about how their results so far can be analysed/interpreted, and what kinds of considerations they need to think about to progress to the next stage of work. Connecting this analysis to the students’ later work in the discussion section of their laboratory report was helpful in making this activity more valuable and relevant to students in the laboratory setting.

Once the discussion has concluded on a suitable rationale for the next step of analysis, the experiment would continue. These discussions intend that this latter process is done with students having better insight into why it is they have chosen particular parameters; that is to say the discussion has supported sense-making of the experimental protocol and related underpinning chemical (and experimental) concepts. Often these kinds of sense-making activities are left to students to determine after class, resulting in significant frustration [4].

### Example 3: Supporting dialogue in the analysis and reporting stages

The above examples have described dialogue actions *within* the laboratory environment. A significant amount of student work — especially at the advanced stages for laboratories relating to analysis of data from instrumentation — is needed in their work done after the laboratory as they continue their analysis and produce a report. This work can be supported by an “exit discussion” dialogue prompt mirroring the “entry discussion” described in Example 1, so that students know what the focus of their post-laboratory work will be.

However, students will likely begin to thoroughly interrogate their data and what it is they are meant to do in their own study time, away from the laboratory. A valuable way to support students as they work through this activity is to use dialogue as mediated through asynchronous discussion forums.

Asynchronous forums have been used to allow students ask questions as they progress through their report. These were initiated after discussions with students highlighted that nearly all of them were spending a very large amount of time on their laboratory reports (15–20 h on a report that was considered would take ~ 3–4 h work). In conversation with students, it emerged that much of this time was spent on figuring out what it was the report required, or just being unsure how to proceed with particular aspects of analysis. We first hosted in-session writing support sessions, but because of timetabling restrictions, we moved these to an online format, using the virtual learning environment discussion forum feature. Each experiment had a dedicated forum, so that as students went through the rotation of experiments through the semester, there was a legacy of information available to them.

In order to prompt students’ use of the forum, they were told that all general questions relating to laboratory reports would only be handled through the forum, with only personal or individual queries permitted by email. Students were also allowed to post anonymously, and asking questions was voluntary. In many cases, students posted a question on behalf of a group of peers, as they worked through the analysis of their work together. Typically, questions related to the various sections of a report.

Queries regarding the introduction related to the purpose and boundaries of what the introduction should contain, and offered an opportunity to re-emphasise the purpose of the introduction to be specific to the students’ experiment, and underpin any subsequent discussion later in their report. For methods, students were often unclear about how much detail to provide, and as well as providing headline guidance, these questions provided useful prompts to share appropriate sections of methods sections in research articles to act as exemplars for students’ writing.

Most queries related to results and discussion. Students were often unsure about how to present their results, how to process them, and what to do if some or all of their data showed the experiment “hadn’t worked”. These queries tended to provide opportunity to reiterate some of the general data analysis guidance provided, and help students troubleshoot their work. Instructors were advised to avoid just providing an answer, but rather to demonstrate scientific thinking in practice, and after providing guidance, offering exit prompts about where the students should go next. General e-moderating guidance [22] advocates not just giving an answer, but rather continuing a conversation. Posts typically ended up with a question, such as “are you able to use this information now to continue your analysis?”, so that the intent of continuing dialogue was clear. Overall the discussion forums leaned into a broader culture of dialogue within the laboratory course, although these observations do point to a need for instructors to consider how students are supported in laboratory report writing more generally [6].

## Conclusions

This article aims to stimulate interest in making explicit the role of dialogue at the heart of learning in the chemistry laboratory. It does so by arguing that learning and understanding are facilitated through the social interactions that the laboratory more easily affords, enabling dialogue between teachers and students, and within student groups. While this potential benefit of laboratory work is valued by students and staff, it is commonly observed that formal dialogue is not embedded in laboratory teaching approaches, and therefore, there is not a consistent level of dialogue happening for all students in all laboratory classes. Often this is because dialogue is assumed, or there is expectancy on students to initiate.

Reflecting on the inclusion of dialogue in the laboratory in our own work highlights that it has led to several benefits. Student feedback for laboratory classes improved dramatically. The laboratory classes described above were often the basis of significant discussion and complaint at staff student liaison committees and other feedback arenas, and the approaches included here helped towards ensuring that students’ work was more directed, and workload was supported (especially in the case of Example 3). Dialogue also helps make explicit the instructors’ thinking, so that students get to observe and interact with scientists and how they explain or work through a problem. As such, dialogue acts as a “wrap-around” for the laboratory experience and its culture, as one of a place where students come to learn in a supportive and immersive environment, where conversation with each other and with instructors was the norm, rather than one where they must struggle through and try to figure out themselves.

Prompts for educators to consider how they could incorporate more explicitly dialogue prompts in their teaching activities are provided. We intend these suggestions to be able to be incorporated into the routine teaching approaches of typical chemistry laboratories, without the need for substantial resource to change.
